# Targeted treatment of brainstem neurohistiocytosis guided by urinary cell-free DNA

**DOI:** 10.1212/NXI.0000000000000299

**Published:** 2016-11-03

**Authors:** David Hunt, Paul Milne, Peter Fernandes, Venetia Bigley, Matthew Collin

**Affiliations:** From MRC Institute of Genetics and Molecular Medicine (D.H.) and Anne Rowling Neurology Clinic, Centre for Clinical Brain Sciences (D.H., P.F.), University of Edinburgh; and Human Dendritic Cell Laboratory (P.M., V.B., M.C.), Institute of Cellular Medicine, Newcastle University, Newcastle upon Tyne UK.

## Abstract

**Objective:**

To identify a treatment-responsive *BRAF*^*V600E*^ mutation in brainstem neurohistiocytosis, where no lesional tissue was readily obtainable, using a cell-free DNA approach.

**Methods:**

Cell-free DNA was extracted from urine and allele-specific PCR for the *BRAF*^*V600E*^ mutation was performed. Response to conventional treatment (corticosteroids and interferon) and targeted treatment with a BRAF inhibitor was assessed by clinical evaluation, gadolinium-enhanced MRI brain scan, and serial testing of urinary cell-free DNA for mutant alleles.

**Results:**

*BRAF*^*V600E*^ mutation could be readily identified in urinary cell-free DNA at an allele frequency of 4.2%. Treatment of Erdheim-Chester disease with corticosteroids and interferon was ineffective and associated with disease progression. Treatment with BRAF inhibitors was associated with clinical improvement and near-complete radiologic remission. Following 6 months of BRAF inhibitor therapy, no enhancing lesions could be detected in the brain and mutant alleles were cleared from the urine.

**Conclusions:**

Analysis of urinary cell-free DNA using allele-specific PCR for *BRAF*^*V600E*^ mutations allows rapid noninvasive identification of a highly treatment-responsive pathway, leading to clinical and radiologic remission of disease. Our case demonstrates that this assay may have a particular role in challenging neurohistiocytosis cases, where attempts at obtaining lesional tissue have failed or are not feasible.

**Classification of evidence:**

This study provides Class IV evidence. This is a single observation study without controls.

Erdheim-Chester disease (ECD) is a neurotropic histiocytic disorder that can present with isolated CNS disease. As such, it is difficult to diagnose and can mimic diseases such as multiple sclerosis^1^ and other neuroinflammatory diseases.^2^ CNS involvement often has a predisposition for deep sites, affecting the brainstem and cerebellum.^3^ Involvement of the CNS confers a poor prognosis with conventional immunomodulatory therapy and is often refractory to treatment.^4^

Recent studies have shown that ECD is a proliferative disorder of the myeloid lineage, with a high frequency of somatic *BRAF*^*V600E*^ mutations.^5^ These findings present the possibility of making a molecular diagnosis and using targeted therapies, sometimes with dramatic benefit.^6–8^ However, identification of such mutations requires the extraction of DNA from lesional tissue. In patients with deep-seated brainstem disease, this may not be an option.^4^ Even when multisystem disease is present, bone biopsy is often highly fibrotic and may not be adequately enriched for somatic mutation or yield DNA of sufficient quality.^3^ A recent report has highlighted the potential utility of cell-free DNA to deliver an accurate molecular diagnosis in histiocytosis.^9^ Cell-free DNA is released by diseased cells into the bloodstream and filtered into the urine, where it can be purified and analyzed for the presence of mutant alleles.^9^

We demonstrate that a highly treatment-responsive *BRAF*^*V600E*^ mutation can be identified by a simple and rapid urine cell-free DNA test in a particularly challenging CNS ECD case, where lesional tissue could not be readily obtained. The identification of the mutation predicted a near-complete response of brain lesions to BRAF inhibition after the failure of conventional therapy.

## CASE REPORT

**Figure 1:**
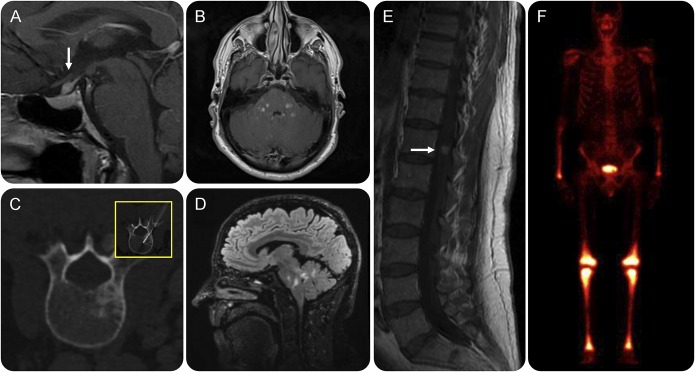
Clinical–radiologic diagnosis of Erdheim-Chester disease (A) Isolated infundibular lesion (arrow) causing diabetes insipidus at presentation. (B) Gadolinium-enhanced MRI scan of the brain; axial sections through pons show multiple enhancing lesions. (C) CT scan of spine shows isolated vertebral sclerotic lesion (biopsy shown was negative for lesional tissue). (D) Sagittal fluid-attenuated inversion recovery MRI of brain shows lesions distributed throughout the pons and cerebellum. (E) Enhancing lesion in cauda equina (white arrow). (F) Bone scan shows characteristic uptake in the long bones (femur, tibia).

A 62-year-old man presented with polydipsia and cranial diabetes insipidus, associated with an isolated pituitary stalk lesion (figure 1A). At presentation, there were no neurologic symptoms and examination was normal. CT scan of the whole body was normal. The patient was kept under clinical and radiologic observation. Over 4 years, he developed gradually progressive bilateral facial numbness, difficulty with visual tracking, and dysarthria. Examination showed evidence of bilateral sensory trigeminal neuropathy, impaired saccadic eye movements, mild dysarthria, and intention tremor. Serial MRI of the brain showed development of multiple persistently enhancing lesions, distributed predominantly throughout the pons and cerebellum (figure 1, B, D, and E). CSF examination was unremarkable, with no oligoclonal bands, no erythrocytes or leukocytes, and normal protein. Bone marrow biopsy was normal. A repeat whole body CT showed a new single sclerotic lesion within the L2 vertebra, with nonspecific findings on biopsy (figure 1C). Bone scan revealed abnormal uptake in distal limb bones (figure 1F). The patient was referred to a national histiocytosis care center and a clinical radiologic diagnosis of ECD was made. The patient received conventional therapy with corticosteroids and interferon-α, with no clinical benefit.

In light of recent evidence suggesting a possible role for BRAF inhibition in the treatment of histiocytic disorders,^6^ options for obtaining biopsy material to look for *BRAF*^*V600E*^ mutation were reviewed. Given the deep location of the brain lesions, the risk of brain biopsy was considered high. Two biopsies of bone revealed no lesional tissue (table e-1 at Neurology.org/nn). The lack of other involved organs presented little option for obtaining fresh lesional material in which to identify targetable kinase mutations. We therefore performed allele-specific *BRAF*^*V600E*^ PCR on urine and peripheral blood.^9^

## METHODS

**Figure 2:**
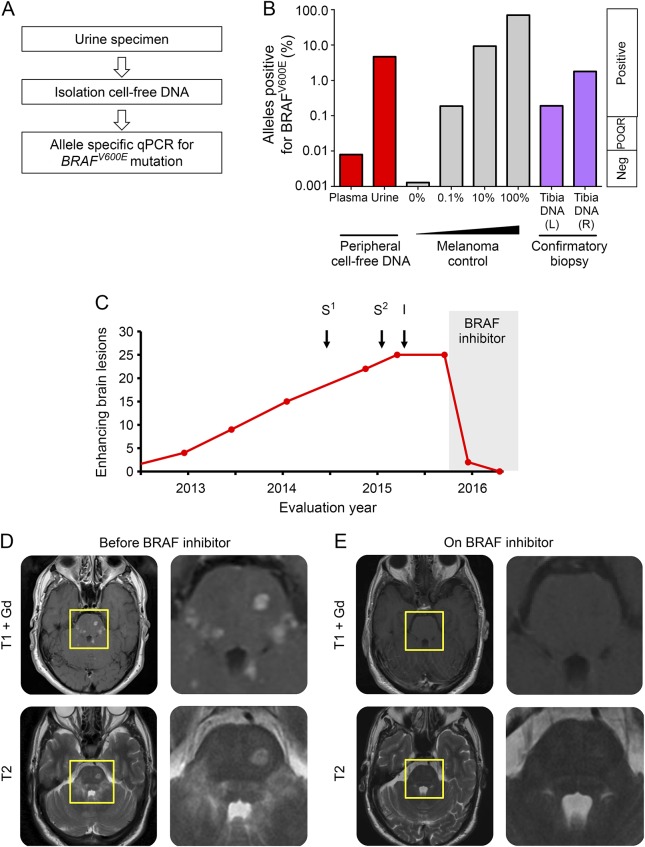
Detection of *BRAF*^*V600E*^ mutation in urine cell-free DNA from a patient with brainstem Erdheim-Chester disease and subsequent response to targeted treatment with dabrafenib (A) Overview of urine cell-free DNA extraction and allele-specific PCR for *BRAF*^*V600E*^ mutation. (B) *BRAF*^*V600E*^ mutation detected by allele-specific PCR from urine cell-free DNA. Red bars: cfDNA extracted from urine and plasma prior to therapy; gray bars: controls 0%, 0.1%, 10%, and 100%; purple bars: DNA from fresh tibial biopsy (see figure 3). (C) Serial evaluation of gadolinium (Gd)–enhancing brain lesions on MRI brain scan, with conventional treatment (S^1/2^ = pulsed IV methylprednisolone 1 g/oral prednisolone 40 mg 2 months; I = recombinant interferon-α: s/c Pegasys 180 μg/wk for 6 months) and BRAF inhibitor (dabrafenib 150 mg bd, ongoing treatment). (D) MRI brain before treatment with dabrafenib: axial T1 Gd-enhanced MRI scan of brain and pons with corresponding T2 sequences (lower). (E) MRI brain after treatment with dabrafenib axial T1 gadolinium-enhanced MRI scan of brain and pons with corresponding T2 sequences (lower). Near-complete resolution of Gd-enhancing lesions is shown, with corresponding reduction in T2 lesion load. POQR = positive outside quantitative range.

Cell-free DNA was extracted from urine and plasma using QIAamp DNA micro and circulating nucleic acid kits (Qiagen; Venlo, Netherlands) and subjected to allele-specific PCR using a *BRAF*^*V600E*^ Taqman Detection Assay (Life Technologies, Nærum,Denmark; figure 2A for overview). DNA from *BRAF*^*V600E*^ melanoma cell line a375 (American Type Culture Collection) diluted into EBV-transformed B cells was used as a positive control. The clinical response to BRAF inhibition was monitored through clinical assessment, serial gadolinium MRI scan of the brain, and detection of mutant alleles in the urine using the urine cell-free DNA assay described above.

## RESULTS

Urine cell-free DNA was positive with 4.2% *BRAF*^*V600E*^ alleles (figure 2B). Peripheral blood mononuclear cell and cell-free DNA gave indeterminate or negative results (figure 2B).

Prior to identification of the *BRAF*^*V600E*^ mutation, the patient was treated with corticosteroids and then recombinant interferon-α therapy (Pegasys 180 μg/wk for 6 months), which is used as first-line therapy for ECD. The patient reported progression of brainstem symptoms during this time and his radiologic appearances worsened (figure 2C).

**Figure 3:**
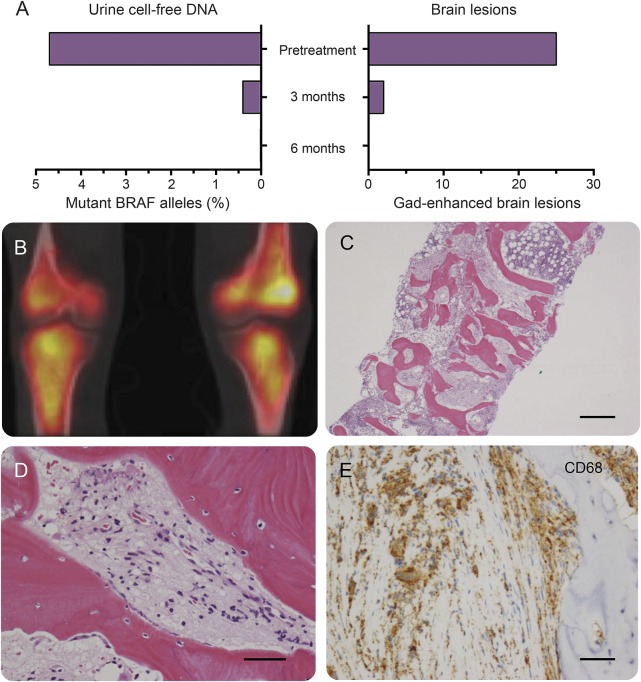
Allele-specific PCR for *BRAF*^*V600E*^ mutation from urine cell-free DNA is a possible biomarker of neurologic disease activity (A) Urine allele-specific PCR was monitored during treatment with BRAF inhibitors and correlated with MRI brain lesion load (gadolinium-enhancing lesions). The presence of the *BRAF*^*V600E*^ mutation was finally confirmed from lesional tissue obtained from surgical tibial biopsy. (B) CT-PET shows disease activity in both tibiae. (C–E) Tibial bone biopsy shows a diffuse infiltrate of foamy mononuclear macrophages (hematoxylin & eosin), which express CD68 and CD163 but not CD1a. DNA was extracted from fresh bone biopsy and was positive for *BRAF*^*V600E*^ mutation. Scale bars: C = 1 mm, D = 100 μm, E = 100 μm.

After detection of *BRAF*^*V600E*^ mutation in urine, the patient was started on an oral BRAF inhibitor (vemurafenib 960 mg bd) but developed moderately severe liver toxicity within 2 weeks of starting treatment and was changed to dabrafenib 150 mg bd. Dabrafenib, an alternative oral BRAF inhibitor, was tolerated well. Within a month of starting treatment with BRAF inhibitors, the patient experienced resolution of dysarthria and saccadic abnormalities. His use of nocturnal desmopressin spray halved. At 3 months of treatment, MRI scan of the brain showed >90% reduction in enhancing lesions with a concomitant 90% reduction in urinary mutant allele count (figures 2, D and E, and 3A). At 6 months of treatment there were no enhancing lesions on MRI brain and *BRAF*^*V600E*^ allele count was undetectable in urine (figure 3A). Clinical and radiologic response has been sustained at 1 year.

Confirmation of the molecular diagnosis was eventually obtained from a surgical tibial bone biopsy that was analyzed fresh (figures 2B and 3, B–E, and table e-1). Histologic findings confirmed the presence of foamy macrophages and CD163+/CD68+/CD1a-cells, consistent with a diagnosis of ECD.

## DISCUSSION

Histiocytic disorders presenting with isolated CNS disease are an important differential diagnosis of multiple sclerosis^10^ and other neuroinflammatory/infiltrative disease.^2,11^ Such presentations are associated with a poor response to standard therapy.^4^ The recent identification of targetable somatic *BRAF* mutations in these disorders highlights the potential benefit for patients with these challenging orphan diseases. However, if this potentially transformative therapeutic approach is to be exploited, lesional tissue is required, which we show here can present a major challenge to clinicians. Even when lesional tissue can be obtained from bone, identification of mutant DNA can fail since bone lesions can be highly fibrotic and cellular content low.^3,9^

We show that a simple rapid noninvasive urine test can guide treatment with a highly effective targeted therapy for deep neurohistiocytosis, where standard therapy has failed. In this case, this test reliably identified a somatic *BRAF*^*V600E*^ mutation where multiple invasive approaches to obtaining lesional tissue had failed. The identification of this mutation identified a near-complete response to BRAF inhibition, in contrast to conventional therapy, which had been associated with relentless disease progression. This neuroradiologic response was observed across all brain MRI sequences used to assess disease activity, including T1 with contrast, T2, and fluid-attenuated inversion recovery, and was associated with clinical improvement.

Importantly, the mutated allele frequency detected in the urine correlated with clinical and radiologic responses, suggesting that this may provide a further noninvasive modality for monitoring response to therapy. This may be particularly important in ECD, where repeat bone biopsy to monitor disease activity may be challenging because of its invasive nature and lack of reliable involvement.^3^

In this case, urine cell-free DNA testing for *BRAF*^*V600E*^ was at least as sensitive as fresh lesional DNA. Our case also shows that although treatment with vemurafenib can cause sufficiently serious liver toxicity to lead to discontinuation of the drug, alternative BRAF inhibitors remain an efficacious option in patients with a somatic *BRAF* mutation.

Therefore, urine cell-free DNA presents an option to diagnose and monitor actionable somatic mutations in neurohistiocytic disorders, especially when lesional tissue is not readily available.

## Supplementary Material

Data Supplement

## GLOSSARY

ECD: Erdheim-Chester disease
