# Supplementary material for: Targeted treatment of brainstem neurohistiocytosis guided by urinary cell-free DNA
Source: Neurol Neuroimmunol Neuroinflamm. 2016 Nov 3;4(1):e299. doi: 10.1212/NXI.0000000000000299 (PMC5096418; doi:10.1212/NXI.0000000000000299)
Supplement: Data Supplement [file supp_4_1_e299__index.html]

Data Supplement 

# Targeted treatment of brainstem neurohistiocytosis guided by urinary cell-free DNA

## Data Supplement

**Files in this Data Supplement:**

- Table e-1 - Microsoft Word file
