# Supplementary material for: Targeted treatment of brainstem neurohistiocytosis guided by urinary cell-free DNA
Source: Neurol Neuroimmunol Neuroinflamm. 2016 Nov 3;4(1):e299. doi: 10.1212/NXI.0000000000000299 (PMC5096418; doi:10.1212/NXI.0000000000000299)
Supplement: Data Supplement [file supp_4.1.e299_Supplementary_Table_1.docx]

|  | Date | Performed by | BRAF mutation status |
| --- | --- | --- | --- |
| Skin biopsy | March 2015 | Dermatology physician | Not tested since no lesional tissue |
| Bone marrow trephine | March 2015 | Haematology physician | Not tested since no lesional tissue |
| Lumbar vertebral biopsy | April 2015 | Interventional neuroradiology | Not tested since no lesional tissue |
| Skin biopsy | May 2015 | Haematology physician | Not tested since no lesional tissue |
| Urine cfDNA | May 2015 | Clinic nurse | Positive |
| Tibial biopsy | August 2015 | Orthopaedic  surgeon | Positive |

**Supplementary Table 1. Evaluation of potential lesional sites for analysing DNA for *BRAF* somatic mutation**
